# Supplementary material for: Knowledge building and vocabulary growth: Assessing the impact of seamless Chinese vocabulary learning for international students
Source: PLoS One. 2025 Feb 24;20(2):e0319285. doi: 10.1371/journal.pone.0319285 (PMC11849872; doi:10.1371/journal.pone.0319285)
Supplement: S1 Appendix — (DOCX) [file pone.0319285.s001.docx]

**S1 Appendix. Target HSK Level 4 words**

Week1

不仅，开玩笑、最好、 共同、幸福、生活、浪漫、缺点、接受、羡慕、爱情、深、性格、幽默、从来

Week2

聚会、互相、逛、短信、联系、平时、讨厌、差不多、麻烦、正好、尽管、真正、困难、无聊、丰富

Week3

约会、印象、准时、判断、信心、紧张、另外、首先、专业、诚实、正式、留、改变、其次、挺

Week4

手忙脚乱、赚、以为、提、调查、原来、提前、计划、保证、提醒、生意、积累、经验、调查、按时

Week5

按照、顺利、感谢、打折、不得不、甚至、奖金、责任、肯定、方法、广告、实际、浪费、标准、消息

Week6

竟然、值得、活动、免费、修理、支持、骗、其中、购物、所有、获得、情况、举办、降低、一分钱一分货

Week7

估计、空气、咳嗽、严重、窗户、动作、后悔、来不及、反对、超过、植物、散步、指、说明、要是、脾气、减肥、辛苦、烦恼

Week 8

巧克力、堵车、心情、愉快、只要、放松、压力、发生、师傅、因此、距离、耐心、态度、到处、缺少

Week9

难道、坚持、主意、饼干、网球、轻松、赢、随便、通过、可是、正确、理想、汗、经历、暂时

Week10

幸福、成功、方向、富、开心、建议、职业、翻译、将来、发展、困、躺、穷、比如

Week11

流利、厉害、学期、猜、来得及、复杂、只好、精彩、无论、杂志、增加、阅读、然而、看法、同时

Week12

规定、死、也许、商量、并且、保护、盐、勺子、解释、对于、使用、引起、误会、节约、相反

Week13

观众、厚、大概、吃惊、遍、来自、申请、有趣、开心、继续、讨论、由、准备、京剧

Week14

打印、复印、道歉、戴、理发、零钱、包子、破、眼镜、租、厨房、房东、乒乓球、饺子、刀

Week15

凉快、热闹、难受、抱、活泼、排队、社会、竞争、受不了、交通、技术、安全、密码、付款、除此之外

Week16

放假、酸、推迟、钥匙、参观、旅行、民族、打扮、究竟、普通话、方言、小吃、收拾、出发、辣
