# Supplementary material for: Knowledge building and vocabulary growth: Assessing the impact of seamless Chinese vocabulary learning for international students
Source: PLoS One. 2025 Feb 24;20(2):e0319285. doi: 10.1371/journal.pone.0319285 (PMC11849872; doi:10.1371/journal.pone.0319285)
Supplement: Attachment — Submitted filename: Response to Reviewers.doc [file pone.0319285.s005.doc]

| Manuscript ID | PONE-D-24-28347 |
| --- | --- |
| Title | Knowledge Building and Vocabulary Growth: Assessing the Impact of Seamless Chinese Vocabulary Learning for International Students |

Dear Reviewer (s) and Editor (s),

First and foremost, we would like to express our utmost gratitude to the Editors and Reviewers for their valuable comments and suggestions and for the effort and time spent in attempting to improve the quality of this article throughout the review process. As such, we have attempted to address all queries and corrections as best as possible.

“Reviewers’ and editor’s comments” have been included (written in black), followed by “Author’s response” (written in red), which explains how the changes have been incorporated or provides further rationale. Some extracts from the paper to show how the reviewers’ comments have been addressed (written in blue). The revised manuscript with tracked changes clearly indicates the location of corrections and adjustments, utilizing the “Track Changes” feature.

We trust we have met the expectations of the Editor and Reviewers. If there are any further concerns or suggestions, please do not hesitate to let us know. Your feedback is immensely valuable to us in improving the quality and rigor of our research.

**Author detailed response:**

**Editor(s):**

**Comment #1:** Please ensure that your manuscript meets PLOS ONE’s style requirements, including those for file naming. The PLOS ONE style templates can be found at

https://journals.plos.org/plosone/s/file?id=wjVg/PLOSOne_formatting_sample_main_body.pdf and <https://journals.plos.org/plosone/s/file?id=ba62/PLOSOne_formatting_sample_title_authors_affiliations.pdf>

**Author response #1:** Thank you for bringing this to our attention. We have carefully reviewed the PLOS ONE style requirements and have made the necessary adjustments to ensure that our manuscript adheres to the prescribed guidelines.

We kindly request your feedback to ensure that the revised manuscript fully meets PLOS ONE’s style and formatting expectations. Thank you for your guidance in improving the quality of our submission.

**Comment #2.** We note that you have indicated that there are restrictions to data sharing for this study. For studies involving human research participant data or other sensitive data, we encourage authors to share de-identified or anonymized data. However, when data cannot be publicly shared for ethical reasons, we allow authors to make their data sets available upon request. For information on unacceptable data access restrictions, please see http://journals.plos.org/plosone/s/data-availability#loc-unacceptable-data-access-restrictions.

Before we proceed with your manuscript, please address the following prompts:

a) If there are ethical or legal restrictions on sharing a de-identified data set, please explain them in detail (e.g., data contain potentially identifying or sensitive patient information, data are owned by a third-party organization, etc.) and who has imposed them (e.g., a Research Ethics Committee or Institutional Review Board, etc.). Please also provide contact information for a data access committee, ethics committee, or other institutional body to which data requests may be sent.

b) If there are no restrictions, please upload the minimal anonymized data set necessary to replicate your study findings to a stable, public repository and provide us with the relevant URLs, DOIs, or accession numbers. Please see http://www.bmj.com/content/340/bmj.c181.long for guidelines on how to de-identify and prepare clinical data for publication. For a list of recommended repositories, please see https://journals.plos.org/plosone/s/recommended-repositories. You also have the option of uploading the data as Supporting Information files, but we would recommend depositing data directly to a data repository if possible. Please update your Data Availability statement in the submission form accordingly.

**Author response #2:** Thank you for highlighting this important aspect of data sharing. We appreciate the journal’s commitment to open science and ethical data management.

Kindly be noted that our study involves sensitive data derived from human participants, which include identifiable information that cannot be fully anonymized due to the context and nature of the collected data. These restrictions have been imposed by our Research Ethics Committee to ensure the privacy and confidentiality of participants, in accordance with institutional and ethical guidelines.

To address requests for data access, we have designated the corresponding author as the primary contact. Therefore, in alignment with PLOS ONE’s data sharing policy, we have uploaded a minimal anonymized data set necessary to replicate our study findings to a stable, public repository. The data is stored on openICPSR with the following details:

Accession Number: openicpsr-211323

Data Link: <https://www.openicpsr.org/openicpsr/workspace?path=openICPSR>

This repository ensures that data is securely hosted and accessible. Requests for access beyond the shared data can be directed to the corresponding author, who will provide guidance based on ethical considerations.

**Revised text:** The revised content has been marked using the “Track Changes” feature on the updated manuscript. For your convenience, it is also presented below for ease of review. We greatly value your feedback and understanding. Thank you very much for your time and assistance.

“Data Availability statement:

The research data have been deposited in the openICPSR repository. For further access, please contact the corresponding author. All requests will be reviewed in accordance with participant privacy and ethical guidelines.”

**Comment # 3:** We note you have included a table to which you do not refer in the text of your manuscript. Please ensure that you refer to Table 7 in your text; if accepted, production will need this reference to link the reader to the Table.

**Author response #3:** Thank you for highlighting this oversight. We acknowledge that Table 7 was not explicitly referred to in the text, which may have caused confusion for readers. We have carefully reviewed the manuscript and have now ensured that Table 7 is directly referenced in the appropriate section. Additionally, we have confirmed that Table 6 is explicitly cited within the text as well. These revisions enhance clarity and effectively guide readers to the relevant data.

**Revised text:** The revised content has been marked using the “Track Changes” feature on the updated manuscript. For your convenience, it is also presented below for ease of review. We greatly value your feedback and understanding. Thank you very much for your time and assistance.

“To further investigate the participating students’ affective experiences, self-efficacy, and motivation within the SCVL environment, the findings reveal that students found it meaningful to connect their in-class learning with out-of-class learning through the use of SCVL (Mean = 4.22), as shown in Table 6.

Table 6. Results of the items on experience in seamless Chinese vocabulary learning.

This connection between learning contexts was supported by qualitative data. For instance, one learner commented:

“The seamless integration of classroom learning with real-life applications through SCVL made learning Chinese more engaging and meaningful.” (L5T, Low, Week 4)

Moreover, students expressed comfort with learning Chinese HSK Level 4 vocabulary through mobile devices outside the classroom, whether at home, on campus, or in other locations (Mean = 4.13). A learner noted:

“I feel very comfortable posting Chinese in the HSK Level 4 Learning Circle and interacting with my classmates. I really enjoy it.” (L6T, Medium, I, Week 8)

Similarly, a medium-performing learner shared a comparable experience:

“At this stage, I feel much better. We can study by ourselves or discuss in the Learning Circle, which is very relaxed and casual.” (L15T, Medium, F, Week 8)

Furthermore, many participants reported that guided seamless learning enhanced their confidence and motivation in learning Chinese vocabulary (Mean = 4.16), as illustrated in Table 7.

This revision ensures that Table 6 & 7 are effectively tied to the narrative, improving both the coherence of the manuscript and the reader’s understanding of the data. Thank you again for pointing out this improvement opportunity!

**Comment # 4:** Please include captions for your Supporting Information files at the end of your manuscript, and update any in-text citations to match accordingly. Please see our Supporting Information guidelines for more information: http://journals.plos.org/plosone/s/supporting-information.

**Author response # 4:** Thank you very much for the reminder and for pointing this out. We have updated the references in our manuscript to use the Numbered reference style as required by the journal.

**Revised text:** The revised content has been marked using the “Track Changes” feature on the updated manuscript.

**Comment # 5：**

Journal Requirements:

Please review your reference list to ensure that it is complete and correct. If you have cited papers that have been retracted, please include the rationale for doing so in the manuscript text, or remove these references and replace them with relevant current references. Any changes to the reference list should be mentioned in the rebuttal letter that accompanies your revised manuscript. If you need to cite a retracted article, indicate the article’s retracted status in the References list and also include a citation and full reference for the retraction notice.

**Author response # 5:** Thank you for your feedback regarding the reference list.

We have carefully reviewed our reference list and made revisions in accordance with the *PLOS ONE* formatting guidelines available at: **[PLOS ONE Submission Guidelines](https://journals.plos.org/plosone/s/submission-guidelines#loc-references) – References.**

We confirm that no retracted articles are cited in our manuscript. Additionally, we have ensured that the reference list is complete and accurate. Kindly refer to the References section in the revised manuscript for your review. Thank you for your time and support.

**Revised text:** The revised content has been marked using the “Track Changes” feature on the updated manuscript.

**Reviewers**

**Reviewer #1:** I think that this is a powerful, informative paper which has the potential to be published and welcomed by the readers of this journal. Overall, the rationale for the paper is clear, the topic, Chinese vocabulary acquisition in higher education, is an area in need of further research, and the authors have designed a learning environment influenced by learning theories, and proven pedagogical learning designs in the form of seamless learning. However, there are some areas that I feel need to be addressed before this study is ready for publication.

The introduction clearly presents the background and some of the aims of the study, but it would be better if you clearly described the main learning tasks and methodology and how they are related to the study. For example, more emphasis needs to be placed on the article writing e.g. authentic, contextual tasks which complement Seamless learning. This needs to be emphasised from the beginning as I feel it is an important aspect of this study. Also, you could mention DBR in the introduction. Perhaps you could discuss why DBR complements seamless learning to strengthen the aims of the study.

The following sentence should be moved from section 3.4 to the introduction to help readers understand the structure of the paper: “However, for the research purpose, the analysis in this paper focuses mainly on the student essay artifacts, which was created using target HSK Level 4 vocabulary.”.

The methodology is mainly well-explained and follows proven steps in DBR but there are a few areas that need further explanation. For example, in section 3.2 please explain how the 240 words were selected. What criteria was used to select the words from the list of 600?

Were the students in China at one university or studying overseas? You mention they were selected from universities from all over China but then took the same course.

There is no explanation of why activity 1 in fig 2 is a social learning activity. It is teacher led activity in the classroom but what is the social aspect? Please explain more about the social learning in the classroom because not all classroom learning can be classed as social learning. For me, it is the activity that makes the learning social.

The class is described as a mixed ability class but for me, the definition of mixed ability would be students from different levels e.g. HSK1, 2, and 3 etc. Perhaps the students should be described as high and low scoring because I think all the students are at the same recognised ability level, HSK 3.

When were the students interviewed? Was it after the study or during the study.

What software did you use to measure the Chinese LFP? Without mentioning the software or process it would be hard for researchers to replicate this procedure.

I do not understand what table 3 shows. Is table 3 an example of the written text above table 3. Perhaps a sentence to explain it would make it clearer. Also, the text box above table 3 needs a caption.

The results and discussion are interesting and seem logical. I just have a couple of suggestions.

Figure 5 needs explaining in more detail. Why did you select data from 6 learners and what is the purpose of showing this data.

Please explain the word cloud in figure 6 in terms of results.

There are a few typos that need correcting. For example, the sentence “Figure Error! No text of specified style in document.” and “figure 4.16” which does not exist.

The manuscript needs page numbers which would also make it easier to give you feedback.

I hope my feedback and suggestions will help you improve what I consider to be a very interesting, well-designed manuscript, and I would be willing to review the final draft.

**Author response:**

Dear Reviewer #1,

Thank you very much for your thoughtful and positive feedback. We are grateful for your recognition of the paper’s potential and the clarity of its rationale. We appreciate your acknowledgment of the importance of researching Chinese vocabulary acquisition in higher education and the effectiveness of the seamless learning environment we have designed.

We also value your constructive suggestions and fully recognize that some areas need further refinement before the study is ready for publication. We are committed to addressing these points and making the necessary revisions to ensure the study’s quality. Your feedback has been invaluable in guiding us through the final steps of this process, and we look forward to implementing the changes to improve the manuscript.

Below, we provide detailed responses to each of your points:

**Reviewer point #1:** The introduction clearly presents the background and some of the aims of the study, but it would be better if you clearly described the main learning tasks and methodology and how they are related to the study. For example, more emphasis needs to be placed on the article writing e.g. authentic, contextual tasks which complement Seamless learning. This needs to be emphasised from the beginning as I feel it is an important aspect of this study. Also, you could mention DBR in the introduction. Perhaps you could discuss why DBR complements seamless learning to strengthen the aims of the study.

The following sentence should be moved from section 3.4 to the introduction to help readers understand the structure of the paper: “However, for the research purpose, the analysis in this paper focuses mainly on the student essay artifacts, which was created using target HSK Level 4 vocabulary.”

**Author response #1:**

Thank you for your constructive feedback. We appreciate your suggestion to clarify and emphasize the main learning tasks and methodology in the introduction, as well as your recommendations for improving the overall clarity of the paper’s structure. We agree that a more explicit description of the article writing tasks, particularly in relation to authentic, contextual tasks and their alignment with seamless learning, would strengthen the introduction and better highlight the study’s aims.

In response to your feedback, we have revised the introduction to provide a clearer explanation of the main learning tasks, focusing on the article writing component and how it complements the seamless learning framework. We have also added a brief discussion of Design-Based Research (DBR) and its relevance to seamless learning to better link the theoretical foundation of the study to the research design.

Regarding the specific sentence about the student essay artifacts, we have moved it from Section 3.4 to the introduction, as per your suggestion, to help readers understand the focus of the study early on.

Here is a revised version of the introduction to incorporate these changes:

**Revised text:** The revised content has been marked using the “Track Changes” feature on the updated manuscript. For your convenience, it is also presented below for ease of review. We greatly value your feedback and understanding. Thank you very much for your time and assistance.

“To address this gap, the present study employs the Design-Based Research (DBR) methodology, which aligns closely with the principles of seamless learning [18]. DBR facilitates the iterative refinement of instructional designs, informed by real-world applications, participant feedback, and contextual requirements [19]. This study places particular emphasis on students’ learning outcomes within a seamless language learning environment, focusing on authentic and contextualised tasks such as essay writing. While the research does not provide an in-depth exploration of instructional design details, it prioritises an evaluation of students’ experiences and measurable learning outcomes [2, 13].

As highlighted by [20], DBR is especially effective in examining learners’ engagement and knowledge-sharing behaviours in mobile seamless learning contexts. In line with this approach, the present study investigates how authentic writing tasks—specifically, student essay artefacts incorporating HSK Level 4 vocabulary—support vocabulary acquisition and application. By integrating real-world and contextualised tasks, this research underscores the importance of seamless learning in bridging formal and informal educational processes to enhance language proficiency.

The analysis focuses primarily on student essay artefacts, which serve as tangible representations of learners’ ability to apply target vocabulary. These artefacts provide valuable insights into the effectiveness of seamless learning environments in fostering both vocabulary acquisition and practical language use. ”

**Reviewer point #2:** The methodology is mainly well-explained and follows proven steps in DBR but there are a few areas that need further explanation. For example, in section 3.2 please explain how the 240 words were selected. What criteria was used to select the words from the list of 600?

**Author response #2:**

Thank you for your valuable feedback and for pointing out the need for further explanation in section 3.2. We acknowledge the importance of providing clarity on the selection process for the 240 words from the list of 600 words and have revised the manuscript accordingly.

In section 3.2, we have expanded the explanation of how the 240 words were selected from the original 600-word list. The selection process was guided by the following criteria:

Relevance to students’ daily lives and academic Contexts: Words were chosen based on their frequency and usefulness in everyday conversations and academic activities relevant to HSK Level 4 learners. For instance, words related to “food,” “travel,” and “health” were prioritized as they are commonly encountered in both formal and informal learning scenarios.

Diversity across vocabulary categories: To ensure a balanced representation, words were selected across key vocabulary categories, including nouns, verbs, adjectives, and adverbs. This categorisation ensures that students are exposed to a range of word types, promoting comprehensive vocabulary development.

Complexity and usage: Words with varying levels of complexity were included, ranging from simple and frequently used terms to more context-specific vocabulary. For example, basic terms like “吃” (eat) and “喜欢” (like) were paired with more specific words like “健康” (health) and “建议” (advice).

Alignment with learning tasks: The selected words were aligned with the themes and contexts of the seamless learning tasks in this study. For instance, writing tasks required students to create sentences and essays using words related to given scenarios, such as describing a weekend activity or writing about a healthy lifestyle.

**Revised text:** The revised content has been marked using the “Track Changes” feature on the updated manuscript. For your convenience, it is also presented below for ease of review. We greatly value your feedback and understanding. Thank you very much for your time and assistance.

“The 240 words (i.e., target HSK Level 4 words) were selected from the official HSK Level 4 vocabulary list of 600 words based on four criteria: relevance to students’ daily lives and academic contexts, diversity across vocabulary categories, complexity and usage, and alignment with the themes of the learning tasks. For example, words like “广告” (advertisement) and “堵车” (traffic jam) were chosen to encourage authentic usage in sentence-making and essay-writing activities.

Additionally, the selected words were curated from the HSK Level 4 syllabus, following the guidance of three expert teachers with over 10 years of experience in teaching CSL. The word selection process also adhered to the “Syllabus of Chinese Language Program for Foreign Learners in Colleges and Universities” (高等学校外国留学生汉语教学大纲, 2002).

This meticulous word selection process aimed to establish a consistent and standardised vocabulary foundation for all CSL learners, facilitating uniformity and comparability in the research study. The full word list can be found in the Appendix for reference.”

**Reviewer point #3:** Were the students in China at one university or studying overseas? You mention they were selected from universities from all over China but then took the same course.

**Author response #3:**

We appreciate your observation regarding the recruitment of students. We sincerely apologize for the oversight in our description. To clarify, the participants were not selected from multiple universities across China, as mistakenly mentioned in the manuscript. Instead, all participants were recruited from one university on the Chinese mainland in 2022. These students were purposively selected from intermediate-level Chinese courses at the university, representing different Chinese language classes. This sampling ensured consistency in their learning level and familiarity with the curriculum, which aligns with the objectives of this study.

**Revised text:** The revised content has been marked using the “Track Changes” feature on the updated manuscript. For your convenience, it is also presented below for ease of review. We greatly value your feedback and understanding. Thank you very much for your time and assistance.

“Participants for this study were purposively selected from intermediate-level Chinese courses at a university (denoted as University A), located on the Chinese mainland, in 2022. They were drawn from different Chinese language classes within the same university to ensure a diverse but comparable representation of learners in the seamless learning program. ”

**Reviewer point #4:** There is no explanation of why activity 1 in fig 2 is a social learning activity. It is teacher led activity in the classroom but what is the social aspect? Please explain more about the social learning in the classroom because not all classroom learning can be classed as social learning. For me, it is the activity that makes the learning social.

**Author response #4:**

We appreciate your observation that not all classroom activities can be classified as social learning, and we agree that it is the activity itself that defines its social aspect. To clarify, Activity 1 involves teacher-facilitated vocabulary learning within a classroom setting, where the social learning aspect arises from its interactive and collaborative nature. During this activity, students engage in discussions to explore word meanings and contexts, ask questions, and share interpretations with their peers. They also observe the teacher’s modeling of vocabulary usage and mimic their peers’ pronunciation, sentence formation, and application of vocabulary in context. Furthermore, students participate in paired or group exercises, practicing vocabulary through short dialogues and group discussions, fostering mutual learning through observation and feedback. As supported by Wong et al. (2016) and Rumjaun & Narod (2020), social learning involves observing and interacting with others, and this interaction is central to Activity 1, providing students with a socially rich environment to refine their language skills. We have revised the manuscript to emphasize these social elements for greater clarity.

**Revised text:** The revised content has been marked using the “Track Changes” feature on the updated manuscript. For your convenience, it is also presented below for ease of review. We greatly value your feedback and understanding. Thank you very much for your time and assistance.

“In-class learning engagement (Activity 1): These activities were facilitated by the teacher during formal lessons, such as identifying and learning new vocabulary from HSK Level 4 textbook passages. Students engaged in discussions to explore word meanings and usage, observed and mimicked the teacher and their peers, and participated in paired or group exercises to practice vocabulary. These activities aimed to prepare students for subsequent activities (2) and (3).”

**Reviewer point #5:** The class is described as a mixed ability class but for me, the definition of mixed ability would be students from different levels e.g. HSK1, 2, and 3 etc. Perhaps the students should be described as high and low scoring because I think all the students are at the same recognised ability level, HSK 3.

**Author response #5:**
Thank you for your observation and for bringing this to our attention. You are absolutely correct, and we apologize for any confusion caused by the use of the term “mixed ability.” All participants were indeed at the same recognized proficiency level, HSK Level 3 (CEFR level B1). To provide a more precise description, we have categorized the participants based on their academic performance rather than implying varying proficiency levels.

Specifically, participants were divided into high- and low-scoring groups based on their HSK Level 3 exam results and their Chinese writing grades obtained from university placement exams. For example, students who scored above 240 out of 300 on the HSK Level 3 exam or achieved a grade of 85% or higher in their placement writing assessments were classified as high-scoring, while those scoring below 180 on the HSK exam or below 70% in their writing assessments were categorized as low-scoring.

This adjustment ensures that the description aligns with the data while highlighting variations in academic performance within the same proficiency level. We will update the manuscript to reflect this clarification and avoid further misunderstandings.

Therefore, a mixed-ability group of 32 international students with high (i.e., students who scored 290 out of 300 on the HSK Level 3 exam and 90% on the university placement writing assessment) to low (i.e., students who scored 180 out of 300 on the HSK Level 3 exam and 65% on the writing assessment) academic performances in Chinese language participated in the study from March to July 2022.

**Revised text:** The revised content has been marked using the “Track Changes” feature on the updated manuscript. For your convenience, it is also presented below for ease of review. We greatly value your feedback and understanding. Thank you very much for your time and assistance.

“Therefore, a mixed-ability group of 32 international students with high (i.e., students who scored 290 out of 300 on the HSK Level 3 exam and 90% on the university placement writing assessment) to low (i.e., students who scored 180 out of 300 on the HSK Level 3 exam and 65% on the writing assessment) academic performances in Chinese language participated in the study. ”

**Reviewer point #6:** When were the students interviewed? Was it after the study or during the study.

**Author response #6:**
Thank you for your careful review and for highlighting this point. The semi-structured interviews were conducted every two weeks during the study. This approach allowed us to track and explore students’ ongoing experiences, perceptions, and challenges with the SCVL program. By collecting real-time feedback, we were able to align with the iterative principles of the Design-Based Research (DBR) methodology and refine the instructional design for the subsequent learning cycles.

Conducting interviews at regular intervals during the study provided valuable insights that not only enriched our understanding of students’ learning processes but also allowed us to make timely adjustments to the SCVL program to enhance its effectiveness.

The interviews were conducted every two weeks and were guided by a set of questions developed based on the research questions, the participants’ seamless Chinese vocabulary learning process, and their responses to the questionnaire. The purpose of these interviews was to track and deeply explore learners’ real-time experiences, emotions, and interactions with the SCVL program.

**Revised text:** The revised content has been marked using the “Track Changes” feature on the updated manuscript. For your convenience, it is also presented below for ease of review. We greatly value your feedback and understanding. Thank you very much for your time and assistance.

“The interviews were conducted every two weeks and were guided by a set of questions developed based on the research questions, the participants’ SCVL process, and their responses to the questionnaire. The purpose of these interviews was to track and deeply explore students’ real-time experiences, emotions, and interactions with the SCVL program.”

**Reviewer point #7:** What software did you use to measure the Chinese LFP? Without mentioning the software or process it would be hard for researchers to replicate this procedure.

**Author response #7:**

Thank you for your careful review and for pointing this out. We apologize for not including the software and detailed process earlier.

We used the software ‘汉语阅读分级指难针’ (available at <https://www.languagedata.net/tester/>) to measure the Chinese Lexical Frequency Profile (LFP). This software classifies and analyzes the frequency of words at each proficiency level in a given text. Specifically, it generates a detailed breakdown of how many words from different levels are present in the text, providing insights into the diversity and complexity of the vocabulary used.

Kindly be noted that the software is available in both **Chinese** and **English** versions. For this study, we used the **Chinese version**, which is specifically designed for analyzing Chinese language texts (See Figure 1). (The English version is used for English corpus analysis.) The software has been widely adopted and validated in prior research for its reliability and accuracy (e.g., Jin et al., 2018; Jin and Lu, 2023).

Figure 1 Screenshot of the “languagedata(**汉语阅读分级指难针)**”

(Friendly Reminder: This software is free to register and use; however, the free version allows for up to five text analyses per day.)

**Revised text:** The revised content has been marked using the “Track Changes” feature on the updated manuscript. For your convenience, it is also presented below for ease of review. We greatly value your feedback and understanding. Thank you very much for your time and assistance.

“Next, the software ‘汉语阅读分级指难针’ (available at https://www.languagedata.net/tester/) was employed to measure the Chinese LFP. This software classifies and analyses the frequency of words at each proficiency level in a given text. Specifically, it generates a detailed breakdown of how many words from different levels are present in the text, providing insights into the diversity and complexity of the vocabulary used. The software has been widely adopted and validated in prior research for its reliability and accuracy [44-45].”

**Reviewer point #8:** I do not understand what table 3 shows. Is table 3 an example of the written text above table 3. Perhaps a sentence to explain it would make it clearer. Also, the text box above table 3 needs a caption.

**Author response #8:**

Thank you for pointing out the need for clarification regarding Table 3 and the text box above it. I appreciate your feedback and have made revisions in the manuscript to ensure better understanding.

In this study, we adapted the Chinese Lexical Frequency Profile (LFP) framework, originally designed for English, to measure Chinese productive vocabulary. The adaptation referenced the structure of the English LFP but replaced its content with the HSK 2.0 Word List(Refer to Table 1), which categorizes vocabulary into six levels: HSK Level 1 (150 words), HSK Level 2 (300 words), HSK Level 3 (600 words), HSK Level 4 (1200 words), HSK Level 5 (2500 words), and HSK Level 6 (5000 words).For example, the essay artifact shown in the text box was written by a CFL student and segmented into words. The segmented text is as follows:

爱情/是/结婚/的/原因/，也/是/配偶/之间/的/安全/和/尊重/，一/个/人/难过/的/时候/，爱人/会/想/办法/为了/让/爱人/很/幸福/，也/接受/对方/的/缺点/很/重要/，因为/每/个/人/都/有/缺点/。 即使/有/问题/，只要/他们/是/一起/就/会/解决/，关系/就/会/加强/更多/，不仅/是/因为/爱/，而且/是/因为/爱/而/产生/的/理解/。

Next, using the software ‘汉语阅读分级指难针’ (Languagedata), this segmented text was analyzed for lexical distribution across HSK levels (Figure 2).

Figure 2 Screenshot of the “HSK Words Distribution Report” (Source: Languagedata)

To further present the adapted Chinese LFP (i.e., the percentage of HSK Level 4 vocabulary distribution ), Table 3 displays the results, including the total word tokens, the number of tokens for each HSK level, and the calculated lexical frequency percentages.

The revised content has been incorporated into the “Revised Manuscript.” as well.

**Revised text:** The revised content has been marked using the “Track Changes” feature on the updated manuscript. For your convenience, it is also presented below for ease of review. We greatly value your feedback and understanding. Thank you very much for your time and assistance.

“In this study, an adapted Chinese LFP was developed by referencing [43] and utilising the HSK 2.0 Word List as its content. This adaptation mirrors the structure of the English LFP but replaces its word family-based approach with a categorisation system aligned with the HSK 2.0 Word List. As shown in Table 1, the HSK 2.0 Word List divides vocabulary into six levels of increasing difficulty, ranging from HSK Level 1 (150 words) to HSK Level 6 (5,000 words).

This adaptation addresses the limitations of traditional English LFPs in analysing Chinese texts by accommodating the unique characteristics of the Chinese language and ensuring relevance to the learners’ proficiency benchmarks. The use of this tailored LFP framework allows for a precise and pedagogically aligned analysis of Chinese vocabulary acquisition within the seamless learning context.

Next, the software ‘汉语阅读分级指难针’ (available at https://www.languagedata.net/tester/) was employed to measure the Chinese LFP. This software classifies and analyses the frequency of words at each proficiency level in a given text. Specifically, it generates a detailed breakdown of how many words from different levels are present in the text, providing insights into the diversity and complexity of the vocabulary used. The software has been widely adopted and validated in prior research for its reliability and accuracy [44-45].

Based on the HSK 2.0 Word List (refer to Table 1), HSK vocabulary distribution and its role in vocabulary growth can be uncovered by examining frequency lists. The adapted Chinese LFP refers to the distribution rates of HSK vocabulary. Therefore, in accordance with the nature of this research, the percentage of HSK Level 4 vocabulary distribution in essay artifacts was employed to measure the productive vocabulary growth in Chinese writing. The calculation of the adapted Chinese LFP is shown below:

LFP = Number of HSK Level 4 word tokens/Total number of word tokens 100% (Adapted based on Laufer and Nation (1995))

Note. LFP refers to the HSK Level 4 vocabulary distribution rate

For example, the essay artifact presented in the text box was written by a CFL student and segmented into words. The segmented text is as follows:

爱情/是/结婚/的/原因/，也/是/配偶/之间/的/安全/和/尊重/，一/个/人/难过/的/时候/，爱人/会/想/办法/为了/让/爱人/很/幸福/，也/接受/对方/的/缺点/很/重要/，因为/每/个/人/都/有/缺点/。 即使/有/问题/，只要/他们/是/一起/就/会/解决/，关系/就/会/加强/更多/，不仅/是/因为/爱/，而且/是/因为/爱/而/产生/的/理解/(English translation: Love is the reason for marriage, and it is also the security and respect between spouses. When one person is feeling down, the partner will find ways to make the other person happy. It is also important to accept each other's flaws because everyone has them. Even when there are problems, as long as they are together, they will solve them, and the relationship will become stronger. This is not only because of love, but also because of the understanding that arises from love)。

The segmented text was then analysed using the software ‘汉语阅读分级指难针’(Languagedata) to calculate the frequency distribution of words at each HSK level (1 to 6). The HSK word distribution report was captured from the software and is shown in Fig 4.

Fig 4. Screenshot of “HSK words distribution report” (Source from Languagedata)

To further illustrate the adapted Chinese LFP (i.e., the percentage of HSK Level 4 vocabulary distribution), Table 3 presents the results, including the total number of word tokens, the number of tokens for each HSK level, and the calculated lexical frequency percentages.

Table 3. Word Distribution Report.

| HSK 2.0 Word list | Number of Word Tokens | Number of Word Types |
| --- | --- | --- |
| HSK Level 1 | 14 | 8 |
| HSK Level 2 | 6 | 4 |
| HSK Level 3 | 8 | 7 |
| HSK Level 4 | 13 | 12 |
| HSK Level 5 | 2 | 2 |
| HSK Level 6 | 1 | 1 |
| Super class words | 16 | 14 |
| In total | 60 | 48 |
| HSK Level 4 vocabulary distribution rate: LFP=Number of HSK Level 4 word tokens/Total number of word tokens 100% | | |
| LFP= 13/60 100%= 21.67% | | |

As shown in Table 3, the total number of word tokens is 60, with 13 of these being HSK Level 4 word tokens, resulting in an LFP of 21.67%.”

**Reviewer point #9:** The results and discussion are interesting and seem logical. I just have a couple of suggestions.

Figure 5 needs explaining in more detail. Why did you select data from 6 learners and what is the purpose of showing this data.

**Author response #9:**

Thank you very much for your kind comments and for pointing out the need for additional detail regarding Figure 5. The data from six learners were selected for the following reasons:

(1) Validation of Individual Impact
    While the previous analysis focused on the vocabulary growth of all 32 participants as a group and three performance categories (low, medium, and high), we wanted to validate whether the SCVL framework also benefits individuals. To achieve this, we selected six learners based on their placement test results, with recommendations from their teachers: two low-performance learners, two medium-performance learners, and two high-performance learners. This allows us to track individual progress and explore the personalized effects of SCVL.

(2) Qualitative Exploration
In addition to quantitative results, analyzing these six learners provided an opportunity to examine their learning experiences, challenges, and perceptions of seamless Chinese vocabulary learning from a qualitative perspective. This approach aims to offer a more comprehensive understanding of SCVL’s impact on different learners.

(3) Highlighting SCVL Details
By showcasing the data from these six learners, we were able to present specific details about how SCVL influences individual vocabulary development, offering insights that complement the group-level analysis.

In a nutshell, the purpose of presenting this data is to provide both quantitative and qualitative evidence of SCVL’s effectiveness, illustrating its potential to support learners across various proficiency levels. We hope this explanation clarifies the selection process and purpose behind the data in Figure 5.

**Revised text:** The revised content has been marked using the “Track Changes” feature on the updated manuscript. For your convenience, it is also presented below for ease of review. We greatly value your feedback and understanding. Thank you very much for your time and assistance.

“These learners were purposively selected based on their HSK Level 3 scores and Chinese writing grades to represent different proficiency levels: two (2) reflecting low performance, two (2) reflecting medium performance, and two (2) reflecting high performance. The aim was to validate the individual impact of SCVL and to gather in-depth qualitative data on learners’ reflections, comments, and perceptions of SCVL, thereby exploring their experiences in greater detail [12]. ”

**Reviewer point #10:** Please explain the word cloud in figure 6 in terms of results.

**Author response #10:**

Thank you for your feedback on the word cloud in Figure 6. In the revised manuscript, we have provided a detailed explanation of the word cloud results to make them clearer.

The word cloud in Figure 6 (It’s Fig 7 now) visually represents the most frequently mentioned words in learners’ reflections and feedback about their experience with SCVL. Larger and more prominently displayed words, such as “helpful,” “improvement,” and “motivated,?” indicate recurring themes in learners’ feedback, highlighting their perceptions of SCVL’s impact.

These results reveal that learners widely acknowledged the benefits of SCVL in improving their vocabulary acquisition and overall Chinese learning. For instance, many learners mentioned that the seamless integration of practice and learning contexts made studying more engaging and practical.

By incorporating qualitative data through the word cloud, we sought to complement the quantitative findings, demonstrating that most learners assuredly gained various benefits from experiencing seamless learning with SCVL. This holistic approach enriches our understanding of SCVL’s effectiveness and learners' experiences.

We hope this revised explanation adequately addresses your comment. Thank you again for your insightful suggestion.

**Revised text:** The revised content has been marked using the “Track Changes” feature on the updated manuscript. For your convenience, it is also presented below for ease of review. We greatly value your feedback and understanding. Thank you very much for your time and assistance.

“The word cloud in Fig. 7 offers a visual representation of the most frequently cited terms in students’ reflections and feedback on their experience with SCVL. Prominent words such as “helpful,” “improvement,” and “motivated” stand out, reflecting recurring themes in the feedback and underscoring students’ perceptions of SCVL’s positive impact. These results indicate that learners widely recognise the benefits of SCVL in enhancing both their vocabulary acquisition and overall Chinese language learning. Many CFL students highlight how the seamless integration of practice with real-world learning contexts made their study experience more engaging and practical, suggesting that this approach not only supported their learning but also fostered greater motivation [25]. This qualitative insight aligns with the overall positive feedback regarding SCVL’s effectiveness in promoting vocabulary development.”

**Reviewer point #11:** There are a few typos that need correcting. For example, the sentence “Figure Error! No text of specified style in document.” and “figure 4.16” which does not exist.

**Author response #11:**

Thank you for pointing out the typos. We apologize for the oversight. The sentence “Figure Error! No text of specified style in document.” has occurred due to a formatting issue, which should have been corrected prior to submission. We have addressed this error and will ensure it is rectified in the revised manuscript. Additionally, the reference to “figure 4.16” was mistakenly included, as it does not exist in the document. We have updated the reference to the correct figure number in the revised version. Your careful review has been greatly appreciated, and we have made the necessary corrections to improve the clarity and accuracy of the manuscript.

**Revised text:** The revised content has been marked using the “Track Changes” feature on the updated manuscript.

**Reviewer point #12:** The manuscript needs page numbers which would also make it easier to give you feedback.

**Author response #12:**

Thank you for your valuable feedback. We apologize for the oversight regarding the absence of page numbers in the manuscript. We have added page numbers in the revised version, which will enhance the readability of the document and make it easier for you to provide feedback. Your attention to detail is much appreciated, and we have made the necessary adjustments.

**Revised text:** The revised content has been marked using the “Track Changes” feature on the updated manuscript.

**Reviewer point #13:** I hope my feedback and suggestions will help you improve what I consider to be a very interesting, well-designed manuscript, and I would be willing to review the final draft.

**Author response #13:**

Thank you for your thoughtful and constructive feedback. We truly appreciate your kind words about the manuscript. Your suggestions have been incredibly helpful in refining and strengthening the work. We are grateful for your willingness to review the final draft, and we will ensure that all necessary revisions are made to enhance the clarity and quality of the manuscript. We look forward to sharing the revised version with you and value your continued support in this process.

**Revised text:** The revised content has been marked using the “Track Changes” feature on the updated manuscript.

Warm regards,

Corresponding Author: ***

**Reviewer #2:** This study is well-designed, with an appropriate and comprehensive literature review. However, the Introduction requires improvement in terms of flow. The concepts are presented in a disjointed manner without a clear connection between them. For example, the first paragraph discusses seamless learning, but the second paragraph shifts abruptly to vocabulary without any transitional elements. Similarly, the discussion transitions to the SCVL approach without making the relationship between these topics clear. Improving the clarity and logical flow of these sections will enhance the paper’s readability.

The paper’s overall formatting also needs revision. Issues such as the error message “Figure Error! No text of specified style in document...” and the lack of clarity in the tables and figures detract from the presentation. The authors should consider consulting other published papers for reference to improve the layout and presentation of visual elements. Furthermore, the statistical results need some formatting adjustments, such as italicizing symbols and ensuring appropriate spacing before and after symbols.

There appears to be a significant issue with the reported standard deviations (SD) in Tables 6 and 7, as the ranges are unusually large, suggesting potential errors. Please verify and report the correct values.

Lastly, it is important to clarify the application of The Lexical Frequency Profile (LFP) in this study. While LFP in English vocabulary studies relies on the word family count method, it is unclear whether the Chinese LFP used here is based on a word list organized by word families. Please provide additional details regarding this aspect to ensure clarity.

**Author response #2**

Dear Reviewer #2,

Thank you very much for your thorough and constructive feedback on our manuscript. We sincerely appreciate the time and effort you have invested in reviewing our work.

We have carefully considered your comments and have made substantial revisions to the manuscript to address the issues you raised. Below, we provide detailed responses to each of your points:

**Reviewer point #1:** However, the Introduction requires improvement in terms of flow. The concepts are presented in a disjointed manner without a clear connection between them. For example, the first paragraph discusses seamless learning, but the second paragraph shifts abruptly to vocabulary without any transitional elements.

**Author response point #1:**

Thank you for highlighting the need to improve the flow of the Introduction. We have carefully revised the section to ensure a seamless transition between the discussion on seamless learning and vocabulary acquisition, with the inclusion of appropriate transitional elements. Below is a revised version with improved coherence and logical progression.

**Revised text:** The revised content has been marked using the “Track Changes” feature on the updated manuscript. For your convenience, it is also presented below for ease of review. We greatly value your feedback and understanding. Thank you very much for your time and assistance.

“Seamless learning, characterised by its ability to integrate formal and informal learning contexts, represents a transformative approach in education [1-2]. .By leveraging mobile and digital technologies, this approach facilitates continuous learning experiences that span across physical and virtual environments, fostering deep engagement and knowledge retention [2-3]. Within this framework, learners are encouraged to integrate real-world practices into their academic development, making seamless learning particularly effective for skill-based disciplines such as language acquisition [2].

In the domain of second language acquisition, vocabulary development plays a pivotal role. Research highlights that contextualised, meaningful learning activities are essential for fostering vocabulary retention and application [4].To effectively foster vocabulary acquisition, a crucial prerequisite is the establishment of professional and authoritative vocabulary syllabi that guide the language learning process [2, 5]. In this regard, China has made significant strides in supporting international students’ Chinese vocabulary acquisition endeavors through the development of the “Syllabus of Chinese Vocabulary and Chinese Characters” in 1991 and the establishment of the “Graded Chinese Syllables, Characters and Words for the Application of Teaching Chinese to the Speakers of Other Languages” as a national standard in 2010.

Furthermore, the Chinese Ministry of Education introduced the Chinese Proficiency Test (HSK or Hanyu Shuiping Kaoshi) as an international standardized test to assess the proficiency of non-native Chinese speakers. The HSK 1.0 Word List was officially launched in 1996, followed by the HSK 2.0 Word List in 2010. Subsequently, in 2021, the HSK 3.0 Word List was updated and partially implemented on July 1, 2021, with the aim of providing comprehensive guidance for future HSK examinations. Table 1 shows the vocabulary size of different new HSK level in HSK 2.0 word list

Table 1. The Vocabulary Size of Different New HSK Level in HSK 2.0 Word list

During this transitional phase, as recommended by numerous scholars [6-7], the HSK 2.0 Word List continues to serve as the vocabulary syllabus for the six global levels of the Chinese Proficiency Examination. For the purpose of this research, the HSK 2.0 Word List was utilized to focus on specific aspects of vocabulary.

The effort taken by the Chinese government to develop the HSK vocabulary lists reflects the significance of the necessity to master Chinese vocabulary, particularly in the field of teaching and learning CFL. However, learning Chinese as a foreign language presents substantial challenges due to the complexities of its writing system and the extensive lexicon required to communicate [8]. Among the many difficulties faced by CFL learners in China, Chinese writing is particularly challenging, often due to students’ limited store of vocabulary [9-10]. Nevertheless, traditional methods of vocabulary teaching often fail to engage learners in ways that connect formal classroom instruction with informal, real-life usage scenarios [2, 11-12]. This gap presents an opportunity to apply the principles of seamless learning to bridge the divide between theory and practice in SLA.

In this context, the Seamless Chinese Vocabulary Learning (SCVL) approach addresses this challenge by embedding vocabulary acquisition into authentic, contextualised activities such as essay writing. SCVL leverages the affordances of mobile technologies and collaborative environments to integrate vocabulary practice with real-world applications [12-13]. By doing so, it not only aligns with the principles of seamless learning but also fosters deeper connections between vocabulary knowledge and practical language use.

Consequently, this study placed great emphasis on enhancing the process of learning Chinese vocabulary for international students by utilising the SCVL approach. The aim was to optimise the learning process and aid international students in comprehending and communicating successfully in written Chinese..

However, despite the substantial research investigating the integration of seamless learning approaches and their impact on language skill acquisition, learning strategies, attitudes, and motivation [14-16], there remains a significant gap in qualitative insights into the authentic experiences, emotions, and challenges faced by CFL students during seamless vocabulary learning. For instance, most empirical studies have focused on quantitative measures, such as pretest-posttest designs, to evaluate the effectiveness of mobile applications or learning systems within a seamless learning framework [14, 16]. Nonetheless, understanding students’ perspectives is essential for identifying both the benefits and limitations of seamless vocabulary learning and for informing instructional strategies [17]. While studies, such as that by [16], have utilized quantitative methods to assess vocabulary acquisition, qualitative insights into learners’ experiences are essential for developing a more nuanced and comprehensive understanding of the impact of seamless learning on vocabulary development.

To address this gap, the present study employs the Design-Based Research (DBR) methodology, which aligns closely with the principles of seamless learning [18]. DBR facilitates the iterative refinement of instructional designs, informed by real-world applications, participant feedback, and contextual requirements [19]. This study places particular emphasis on students’ learning outcomes within a seamless language learning environment, focusing on authentic and contextualised tasks such as essay writing. While the research does not provide an in-depth exploration of instructional design details, it prioritises an evaluation of students’ experiences and measurable learning outcomes [2, 13].

As highlighted by [20], DBR is especially effective in examining learners’ engagement and knowledge-sharing behaviours in mobile seamless learning contexts. In line with this approach, the present study investigates how authentic writing tasks—specifically, student essay artefacts incorporating HSK Level 4 vocabulary—support vocabulary acquisition and application. By integrating real-world and contextualised tasks, this research underscores the importance of seamless learning in bridging formal and informal educational processes to enhance language proficiency.

The analysis focuses primarily on student essay artefacts, which serve as tangible representations of learners’ ability to apply target vocabulary. These artefacts provide valuable insights into the effectiveness of seamless learning environments in fostering both vocabulary acquisition and practical language use. ”

**Reviewer point #2:** Similarly, the discussion transitions to the SCVL approach without making the relationship between these topics clear. Improving the clarity and logical flow of these sections will enhance the paper’s readability.

**Author response point #2:**

Thank you for your observation regarding the lack of clarity in transitioning to the SCVL approach.

**Revised text:** The revised content has been marked using the “Track Changes” feature on the updated manuscript.

**Reviewer point #3:** The paper’s overall formatting also needs revision. Issues such as the error message “Figure Error! No text of specified style in document...” and the lack of clarity in the tables and figures detract from the presentation.

**Author response point #3:**

Thank you for highlighting the formatting issues related to figures and tables in the manuscript. We have thoroughly reviewed the document to address these concerns and made the necessary corrections to improve the clarity, presentation, and professionalism of the visual elements.

**Revised text:** The revised content has been marked using the “Track Changes” feature on the updated manuscript.

**Reviewer point #4:** Furthermore, the statistical results need some formatting adjustments, such as italicizing symbols and ensuring appropriate spacing before and after symbols.

**Author response point #4:** We appreciate your meticulous review of our manuscript and for pointing out the need for formatting adjustments in the statistical results. We have carefully reviewed the statistical notations and revised them to conform to standard formatting guidelines. Symbols such as p, t, and F have been italicized, and appropriate spacing has been added before and after these symbols for clarity and consistency.

**Revised text:** The revised content has been marked using the “Track Changes” feature on the updated manuscript.

**Reviewer point #5:** There appears to be a significant issue with the reported standard deviations (SD) in Tables 6 and 7, as the ranges are unusually large, suggesting potential errors. Please verify and report the correct values.

**Author response point #5:** We greatly appreciate your detailed review and for pointing out the unusually large ranges in the reported standard deviations (SD) in Tables 6 and 7. Upon reevaluating the data, we identified an error in our calculations and formatting. The correct SD values have been reviewed and updated to ensure accuracy.

We apologize for any confusion caused by this error and have taken steps to verify all statistical data thoroughly. The revised tables with accurate SD values are included in the updated manuscript. Thanks again!

**Revised text:** The revised content has been marked using the “Track Changes” feature on the updated manuscript. For your convenience, it is also presented below for ease of review. We greatly value your feedback and understanding. Thank you very much for your time and assistance.

**Table 6. Results of the items on experience in SCVL.**

| **Item** | **Mean score** | **SD** |
| --- | --- | --- |
| I am comfortable with Chinese HSK Level 4 vocabulary learning through my mobile device outside of the classroom (at home, on campus, and other places, etc.). | 4.13 | 0.70 |
| It is interesting to learn Chinese HSK Level 4 vocabulary under seamless learning after class hours. | 3.94 | 0.83 |
| I am happy to share content in Chinese about my daily life on social media platform. | 4.03 | 0.85 |
| It is meaningful to connect my in-class learning to my out-of-class learning of Chinese. | 4.22 | 0.60 |

Table 7. Results of the items on self efficacy and motivation in SCVL.

| **Item** | **Mean score** | **SD** |
| --- | --- | --- |
| I am able to learn Chinese HSK Level 4 vocabulary from in class formal learning to out-of-class informal learning through the use of mobile devices and social media platforms. | 4.03 | 0.64 |
| Informal learning guided by teaching instructor in seamless Chinese HSK Level 4 vocabulary learning can support my formal classroom learning. | 4.25 | 0.71 |
| Guided informal learning under seamless Chinese HSK Level 4 vocabulary learning after class hours is important for me to improve the mastery of Chinese vocabulary. | 4.19 | 0.80 |
| Guided seamless learning gives me confidence and motivation in learning Chinese vocabulary. | 4.16 | 0.76 |

We thank you once again for highlighting this issue and allowing us to correct and improve the manuscript. We hope the revised version meets the required standards and look forward to your further feedback.

**Reviewer point #6:** Lastly, it is important to clarify the application of The Lexical Frequency Profile (LFP) in this study. While LFP in English vocabulary studies relies on the word family count method, it is unclear whether the Chinese LFP used here is based on a word list organized by word families. Please provide additional details regarding this aspect to ensure clarity.

**Author response point #6:**

Thank you for pointing out the need to clarify the application of The Lexical Frequency Profile (LFP) in this study.

The Lexical Frequency Profile (LFP) in this study was adapted to suit the unique characteristics of Chinese vocabulary. While traditional LFPs in English studies rely on the word family count method, the Chinese LFP used here differs in its organisation and content. Specifically, this study developed an adapted Chinese LFP by referencing the framework provided by [43] and incorporating the HSK 2.0 Word List as its foundation.

Unlike the word family-based categorisation used in English LFPs, the Chinese LFP is structured around the HSK 2.0 Word List, which categorises vocabulary into six levels of increasing complexity:

HSK Level 1: 150 words

HSK Level 2: 300 words

HSK Level 3: 600 words

HSK Level 4: 1,200 words

HSK Level 5: 2,500 words

HSK Level 6: 5,000 words

This adaptation ensures that the LFP is both linguistically relevant and contextually appropriate for analysing Chinese texts. It resolves limitations associated with the traditional English LFP model, particularly in accommodating the structural and semantic differences inherent in the Chinese language.

By adopting this framework, the study effectively aligns the LFP analysis with the pedagogical standards outlined in the HSK 2.0 Word List, providing a reliable basis for evaluating learners’ vocabulary proficiency. Table 1 (referenced in the manuscript) summarises the categorisation criteria and word counts at each HSK level.

**Revised text:** The revised content has been marked using the “Track Changes” feature on the updated manuscript. For your convenience, it is also presented below for ease of review. We greatly value your feedback and understanding. Thank you very much for your time and assistance.

“In this study, an adapted Chinese LFP was developed by referencing [43] and utilising the HSK 2.0 Word List as its content. This adaptation mirrors the structure of the English LFP but replaces its word family-based approach with a categorisation system aligned with the HSK 2.0 Word List. As shown in Table 1, the HSK 2.0 Word List divides vocabulary into six levels of increasing difficulty, ranging from HSK Level 1 (150 words) to HSK Level 6 (5,000 words).

This adaptation addresses the limitations of traditional English LFPs in analysing Chinese texts by accommodating the unique characteristics of the Chinese language and ensuring relevance to the learners’ proficiency benchmarks. The use of this tailored LFP framework allows for a precise and pedagogically aligned analysis of Chinese vocabulary acquisition within the seamless learning context. ”

Thank you again for your thoughtful and constructive feedback. We truly appreciate your kind words about the manuscript. Your suggestions have been incredibly helpful in refining and strengthening the work. We are grateful for your willingness to review the final draft, and we will ensure that all necessary revisions are made to enhance the clarity and quality of the manuscript. We look forward to sharing the revised version with you and value your continued support in this process.

Best regards,

Corresponding Author: ***

Overall, we would like to express our heartfelt thanks to the editors and reviewers for their insightful feedback, which has greatly enriched the quality of our manuscript.

We are confident that the manuscript now aligns with the high standards expected for publication in this esteemed journal. We sincerely appreciate your understanding and interest in our work. We believe this study will greatly interest educators, researchers, and practitioners in the fields of language education and educational technology. We are hopeful that our contributions will be well received by the academic community.

Thank you once again for your time and effort in reviewing our manuscript.

Sincerely,

Corresponding Author: ***
