# Supplementary material for: Knowledge building and vocabulary growth: Assessing the impact of seamless Chinese vocabulary learning for international students
Source: PLoS One. 2025 Feb 24;20(2):e0319285. doi: 10.1371/journal.pone.0319285 (PMC11849872; doi:10.1371/journal.pone.0319285)
Supplement: Attachment — Submitted filename: PONE-D-24-28347 review.docx [file pone.0319285.s002.docx]

I think that this is a powerful, informative paper which has the potential to be published and welcomed by the readers of this journal. Overall, the rationale for the paper is clear, the topic, Chinese vocabulary acquisition in higher education, is an area in need of further research, and the authors have designed a learning environment influenced by learning theories, and proven pedagogical learning designs in the form of seamless learning. However, there are some areas that I feel need to be addressed before this study is ready for publication.

The introduction clearly presents the background and some of the aims of the study, but it would be better if you clearly described the main learning tasks and methodology and how they are related to the study. For example, more emphasis needs to be placed on the article writing e.g. authentic, contextual tasks which complement Seamless learning. This needs to be emphasised from the beginning as I feel it is an important aspect of this study. Also, you could mention DBR in the introduction. Perhaps you could discuss why DBR complements seamless learning to strengthen the aims of the study.

The following sentence should be moved from section 3.4 to the introduction to help readers understand the structure of the paper: “However, for the research purpose, the analysis in this paper focuses mainly on the student essay artifacts, which was created using target HSK Level 4 vocabulary.”

The methodology is mainly well-explained and follows proven steps in DBR but there are a few areas that need further explanation. For example, in section 3.2 please explain how the 240 words were selected. What criteria was used to select the words from the list of 600?

Were the students in China at one university or studying overseas? You mention they were selected from universities from all over China but then took the same course.

There is no explanation of why activity 1 in fig 2 is a social learning activity. It is teacher led activity in the classroom but what is the social aspect? Please explain more about the social learning in the classroom because not all classroom learning can be classed as social learning. For me, it is the activity that makes the learning social.

The class is described as a mixed ability class but for me, the definition of mixed ability would be students from different levels e.g. HSK1, 2, and 3 etc. Perhaps the students should be described as high and low scoring because I think all the students are at the same recognised ability level, HSK 3.

When were the students interviewed? Was it after the study or during the study.

What software did you use to measure the Chinese LFP? Without mentioning the software or process it would be hard for researchers to replicate this procedure.

I do not understand what table 3 shows. Is table 3 an example of the written text above table 3. Perhaps a sentence to explain it would make it clearer. Also, the text box above table 3 needs a caption.

The results and discussion are interesting and seem logical. I just have a couple of suggestions.

Figure 5 needs explaining in more detail. Why did you select data from 6 learners and what is the purpose of showing this data.

Please explain the word cloud in figure 6 in terms of results.

There are a few typos that need correcting. For example, the sentence “Figure Error! No text of specified style in document.” and “figure 4.16” which does not exist.

The manuscript needs page numbers which would also make it easier to give you feedback.

I hope my feedback and suggestions will help you improve what I consider to be a very interesting, well-designed manuscript, and I would be willing to review the final draft.
